# Vertical locomotion improves horizontal locomotion: effects of climbing on gait and other mobility aspects in Parkinson’s disease. A secondary analysis from a randomized controlled trial

**DOI:** 10.1186/s12984-024-01363-4

**Published:** 2024-04-27

**Authors:** Agnes Langer, Clint Hansen, Dominik Roth, Agnes Santer, Anna Flotz, Jakob Gruber, Laurenz Wizany, Sebastian Hasenauer, Rochus Pokan, Peter Dabnichki, Marco Treven, Sarah Zimmel, Michaela Schmoeger, Ulrike Willinger, Lucia Gassner, Christof Brücke, Walter Maetzler, Heidemarie Zach

**Affiliations:** 1 Department of Neurology, Medical University of Vienna, Waehringerstrasse 18-21, 1090 Vienna, Austria; 2 Department of Neurology, University Hospital Schleswig-Holstein and Kiel University, 24105 Kiel, Germany; 3 Department of Emergency Medicine, Medical University of Vienna, 1090 Vienna, Austria; 4 Department of Sport Physiology, Institute of Sports Sciences, University of Vienna, 1090 Vienna, Austria; 5 School of Engineering, RMIT University, 3000 Melbourne, VIC Australia

**Keywords:** Parkinson’s disease, Gait, Mobility, Sport climbing, Exercise

## Abstract

**Background:**

In the Climb Up! Head Up! trial, we showed that sport climbing reduces bradykinesia, tremor, and rigidity in mildly to moderately affected participants with Parkinson’s disease. This secondary analysis aimed to evaluate the effects of sport climbing on gait and functional mobility in this cohort.

**Methods:**

Climb Up! Head Up! was a 1:1 randomized controlled trial. Forty-eight PD participants (Hoehn and Yahr stage 2–3) either participated in a 12-week, 90-min-per-week sport climbing course (intervention group) or were engaged in regular unsupervised physical activity (control group). Relevant outcome measures for this analysis were extracted from six inertial measurement units placed on the extremities, chest, and lower back, that were worn during supervised gait and functional mobility assessments before and after the intervention. Assessments included normal and fast walking, dual-tasking walking, Timed Up and Go test, Instrumented Stand and Walk test, and Five Times Sit to Stand test.

**Results:**

Compared to baseline, climbing improved gait speed during normal walking by 0.09 m/s (p = 0.005) and during fast walking by 0.1 m/s. Climbing also reduced the time spent in the stance phase during fast walking by 0.03 s. Climbing improved the walking speed in the 7-m- Timed Up and Go test by 0.1 m/s (p < 0.001) and the turning speed by 0.39 s (p = 0.052), the speed in the Instrumented Stand and Walk test by 0.1 m/s (p < 0.001), and the speed in the Five Times Sit to Stand test by 2.5 s (p = 0.014). There was no effect of sport climbing on gait speed or gait variables during dual-task walking.

**Conclusions:**

Sport climbing improves gait speed during normal and fast walking, as well as functional mobility in people with Parkinson’s disease.

*Trial registration* This study was registered within the U.S. National Library of Medicine (No: NCT04569981, date of registration September 30th, 2020)

**Supplementary Information:**

The online version contains supplementary material available at 10.1186/s12984-024-01363-4.

## Background

Parkinson’s disease (PD) is a neurodegenerative movement disorder whose cardinal clinical symptoms, i.e. bradykinesia, rigidity, and tremor, as well as postural instability, are mainly caused by the gradual degeneration of dopaminergic neurons and projections in the basal ganglia. The loss of dopaminergic neurons in the substantia nigra pars compacta leads to dopamine depletion in the striatum. The resultant inhibitory output through the basal ganglia pathways to the subcortical and cortical areas, which control voluntary movement control, results in the characteristic slow movements, such as hypokinetic gait [1, 2]. The basal ganglia play an essential role in the supraspinal locomotor network [3, 4], where gait is regulated by modulating rhythmic step patterns originating from spinal central pattern generators [5] to meet external conditions. This circuit, which allows for adaptive gait [4], is disrupted in PD due to the impaired basal ganglia component of the locomotor network. Gait disorders in PD are thus multifactorial and determined by a combination of bradykinesia, reduced postural control [6, 7], and impaired gait variability [3].

Walking under dual-task conditions (i.e., performing two tasks simultaneously) is also known to be affected in PD participants [8–11], owing to deficits in both the motor domain and higher cognitive function associated with the disease [12, 13].

Not only does the gait dysfunction in PD lead to more frequent falls [14] with subsequent injuries and hospital stays [15], but it also is one of the most debilitating symptoms of PD [16], depriving affected individuals of their independent mobility and ultimately their autonomy [17]. Identifying methods to alleviate impaired gait in PD is a crucial task that comes with its own set of challenges, seeing as pharmacological interventions [17] have yielded mixed results and Deep Brain Stimulation has shown only modest effects on gait dysfunction [18]. Physical exercise can not only alleviate the cardinal PD motor symptoms [19] and improve quality of life overall [20] but also improve gait, as shown in several meta-analyses on various types of exercise, but its effectiveness is highly dependent on patients’ commitment to long-term training [21]. PD patients face various obstacles to exercise adherence such as motor symptoms, depression, pain, and fatigue [22], therefore finding appealing sports to maximise exercise’s therapy effects is critical for sustained benefits in this population [23]. Conventional physical therapy [24], targeted gait exercises [25, 26], music-based exercise [27, 28], treadmill training [29], and even self-guided exercise [30] improve gait in PD. There is also evidence that dual-task-targeted training improves walking under this condition in PD [31–34]. Physical exercise can also improve functional mobility aspects, such as transitions (standing up and sitting down), standing, and turning, sometimes collectively referred to as “functional mobility” by some authors and most commonly assessed with the Timed Up and Go test (TUG) [35–37].

As mentioned above, it is important to widen the range of sports for PD patients that are both effective against gait dysfunction and engaging to ensure long-term adherence. Sport climbing is a promising modality for PD, as it is a safe sport [38] that is not only known to increase cardiorespiratory and muscular fitness parameters but also cognition in healthy adults [39–41]. It also effectively improves balance and coordination in multiple sclerosis [42, 43] and ataxia [44], and shows favourable adherence rates [45, 46]. Most importantly, sport climbing reduces motor symptoms of PD by as much as 13 points (34%) on the MDS-UPDRS III scale, as shown in our recent randomized trial [47]. Whether sport climbing also has an effect on gait and functional mobility in PD, has not been studied before and is the subject of this secondary analysis. The vertical nature of climbing demands a high degree of precision, balance, and coordination, all of which are essential for improving gait, potentially transferring these skills to improve horizontal gait function and reduce fall risk in patients.

We hypothesized an improvement of gait parameters (i) in single-tasking due to the known positive effect of sport climbing on bradykinesia and rigidity, (ii) in dual-task walking due to the cognitively demanding nature of sport climbing [48–51], and (iii) in functional mobility aspects such as standing up, standing and turning due to reduction of PD motor symptoms [47].

## Methods

### Standard protocol approvals, registrations, and patient consent

This study is part of the “Climb up! Head up” project, which was approved by the ethical committee of the Medical University of Vienna (No. 1774/2012) and registered with the US National Library of Medicine (No: NCT04569981). All participants provided written informed consent before being included in the trial.

### Study design and participants

The detailed study protocol has been published recently [47]. In brief, this was a single-center, randomized semiblind trial. We included participants with mild to moderate idiopathic PD (Hoehn & Yahr [H&Y] stage 2–3), diagnosed according to the UK Brain Bank criteria [52] without prior climbing experience and recruited them from the Medical University of Vienna Movement Disorders Clinic. Participants were deemed ineligible for the trial if they had a history of stroke, severe orthopedic problems, severe visual or hearing problems, and significant cognitive impairment (Mini-Mental State Examination score [MMSE] < 24 [53]). As this was a secondary analysis of a randomized controlled trial, there was no formal sample size calculation for the outcome presented here.

### Interventions

The intervention group, “sport climbing group” (SC), followed a 12-week, 90-min-per-week supervised sport climbing course in an indoor climbing hall, with an instructor-to-patient ratio of 1: 3–4. The climbing style chosen for the study was toprope climbing, which is the most common style at indoor climbing walls. It requires a “belayer”, i.e., a person standing on the ground who secures the rope holding the climber. The rope runs from the belayer to the climber via carabiners connected to an anchor system at the top of the climbing wall.

The control group, the “unsupervised training group” (UT), independently followed the “European Physiotherapy Guidelines for Parkinson’s disease” and World Health Organization (WHO) recommendations for an active lifestyle for 12 weeks [54, 55]. The recommendations advise participants to perform moderate aerobic physical activities for 150 min per week, strength training twice a week, and balance exercises three times per week [54, 55]. We instructed the participants to complete a training log and performed telephone follow-up calls every seven to ten days. We discouraged changes in dopaminergic medication and deep brain stimulation settings throughout the study period whenever possible to minimize a confounding effect. However, if adjustments were necessary to ensure the participants’ optimal treatment, this was not considered an exclusion criterion.

### Procedures

We performed all assessments at baseline and after the end of the 12-week intervention in the participants’ subjectively best ON-state between 1 and 2h after their dopaminergic morning medication. All raters performing the clinical evaluation were blinded to the participants’ group allocations and were trained in the use of all assessment tools. Gait and functional mobility tests took place in a two-meter-wide, obstacle-free hallway in the outpatient clinic of the Department of Neurology. The start and the end of the walkway were clearly marked on the ground. For the correct performance of each task, standardised verbal instructions were given, as well as a start command. We did not perform practice trials. The sequence of the individual trials was the same for all participants. The participants were able to sit and rest at any time between the tasks as often and as long as necessary.

### Outcome measures

Performance-based tests and inertial sensors were used to quantify gait.

The test began with the dual-task walking sequence, which was divided into a “motor dual task” and a “cognitive dual task”. Both forms of dual-task walking are important measures of mobility in Parkinson’s disease [12, 56]. For the “motor dual task”, participants were given a clipboard with a sheet of paper on which a grid of 32 squares was pre-printed. The aim was to make a cross in each of the given boxes as quickly as possible. For the “cognitive task”, participants performed ten sequential subtractions from a three-digit number in steps of seven (or three, if seven was too difficult). The times taken to complete the tasks were measured in seconds.

The participants began the test sequence by performing the components of the dual-task test separately as a single task: first, they completed the motor and cognitive tasks while standing. The participants then walked 20 m as fast as possible (fast walking) at their preferred speed (normal walking). The tasks were then combined into two dual-task tasks: Walking as fast as possible while crossing out boxes (“motor dual-task walking”) and walking as fast as possible while subtracting (“cognitive dual-task walking”). No direction was given on prioritizing either of the concurrent tasks. Gait speed was measured with a stopwatch. Then the participants performed the TUG [57], a test used to measure mobility in PD [58] under single and dual-tasking conditions, the latter of which is especially sensitive for identifying a risk of falls [59, 60]. For the TUGs, the participants had to stand up from a chair, walk a predetermined distance, return to the chair, and sit down again. The test was performed twice with a seven-meter distance (7 m-TUG), starting first with the left leg and then with the right leg, and once with a three-meter distance (3 m-TUG), starting with their preferred foot. The time between standing up and sitting back down was measured in seconds. The dual-task-TUG consisted of the 3 m-TUG (standing up from a chair, walking 3 m, walking back, and sitting down), with an additional cognitive task in the form of simultaneous mental arithmetic (subtractions in increments of three from 202).

The participants then performed the Instrumented Stand and Walk Test (ISAW) [61], which is a compound measure of gait and balance [62] and can predict the risk of falls [63]. For the ISAW, participants first stood still for 30 s, then walked seven meters, turned around, and walked seven meters back to the starting position. The test was performed twice, each time starting with a different leg, and the time was measured starting after the 30-s stand-still. For the statistical calculation, both rounds were computed together. The final test was the Five Times Sit to Stand test (5TSTS), which was designed to measure mobility, balance, and leg strength in the elderly and PD patients [64–66] and correlates with the risk of falls in PD [64]. The 5TSTS consisted of standing up from a chair without the help of their arms and sitting back down as fast as possible. The time needed to complete five consecutive cycles of standing up and sitting back down was measured in seconds.

Inertial measurement unit (IMU) -derived gait parameters were obtained during all gait and functional mobility tests using a validated [62, 67, 68] movement analysis system (Mobility Lab^®^, APDM Inc., OR, USA) consisting of six inertial recording units triaxial accelerometers, gyroscopes and magnetometers that measure angular displacements and velocities of trunk and limb movements during walking. The IMUs were applied in a standardised fashion to the ankles, wrists, lumbar spine, and chest. Using established company-provided and validated algorithms, we extracted the following quantitative gait parameters: cadence (number of steps/meter), step time, step time variability, stride time, stance time, swing time, double limb support time, double limb support time variability, stride time asymmetry. For the 5TSTS, Stand-to-Sit Angle (degrees), Stand-to-Sit Duration, and Stand-to-Sit flexion speed, and Stand-to-Sit extension speed were measured. For the TUG tests and the ISAW, the duration of turns and angular velocity were measured.

For an overview of the gait and functional mobility tests, see Table 1.

**Table 1:** Overview of gait and other mobility tests

	Additional dual-task tests		Inertial measurement unit (IMU)
	Motor dual-task	Cognitive dual-task	
Gait tests			
20 m normal walking			Y
20 m fast walking	Y	Y	Y
Functional mobility tests			
Instrumented stand and walk			Y
Timed up-and-go		Y	Y
Five times sit-to-stand			Y

Y, yes (indicates that additional dual task tests or inertial measurement units were applied during this test)

### Statistical analysis

The results were tabulated by baseline vs. after 12 weeks and by group (SC vs. UT). We then separately calculated absolute mean differences for each group between baseline and after 12 weeks with robust 95% confidence intervals. We formally tested the group assignment’s influence on gait parameters using a linear regression model to analyse differences between groups. The dependent variable was the mean score of speed and IMU-derived gait characteristics after 12 weeks, and the indicator-covariate was the intervention group assignment. We present both coefficients and p values generated from the covariate’s t-statistic. The significance level was set at a two-sided p-value < 0.05. We did not account for multiple testing because this is a hypothesis-generating, exploratory study. We used JASP 0.1.6.3 for all analyses [69].

## Results

### Baseline characteristics

We included 48 participants who met the inclusion criteria. There were two drop-outs in SC, one due to a loss of motivation and one due to an unrelated, newly diagnosed malignant neoplasm. Baseline characteristics were similar across both groups (see Table 2).

**Table 2:** Baseline characteristics of participants

	SC	UT
	(n = 24)	(n = 24)
Sex: female/male (%)	10/14 (42/58)	8/16 (33/67)
age, mean (range)	65 (45–78)	64 (49–78)
Disease duration, months since diagnosis (range)	77 (2–144)	63 (2–180)
MDS-UPDRS-III score, mean (SD)	37.9 (10.9)	34.2 (14.2)
Hoehn and Yahr stage: 2/3 (%)	20/4 (83/17)	22/2 (92/8)
LEDD, mg (range)	554 (200–1365)	609 (0–1464)
Patients not on dopaminergic therapy, n (%)	0 (0)	1 (4)
Patients with Deep Brain Stimulation, n (%)	1 (4)	1 (4)
Mini Mental State Examination score, mean (SD)	29 (1)	29 (1)

SC, sport climbing group; UT, unsupervised physical training group; Hoehn and Yahr stage (score 0–5); MDS-UPDRS-III, motor part of the Movement Disorder Society-Sponsored Revision of the Unified Parkinson’s Disease Rating Scale (score 0–132; lower scores indicate milder symptoms); LEDD, levodopa equivalent daily dose per day; MMSE, Mini-Mental State Examination (score 0–30; higher scores indicate better functioning). Data are mean (range, percentage) unless indicated otherwise. SD, standard deviation

### Main result

Additional file 1: Table S1 shows the mean values for gait variables collected during normal and fast walking, dual-task walking, and functional mobility tests at baseline and after the intervention.

### Normal walking

After the intervention, the SC exhibited an increase in walking speed by 0.09 m/s, which was significant (95% CI [0.04–0.14], p = 0.003).

Additionally, there was a reduction in step time asymmetry by 0.02 s (95% CI [− 0.03 to 0.01], p = 0.008). No significant changes were observed in other IMU-derived gait parameters.

The UT did not show significant changes in walking speed or IMU-derived gait parameters.

**Fig. 1:**
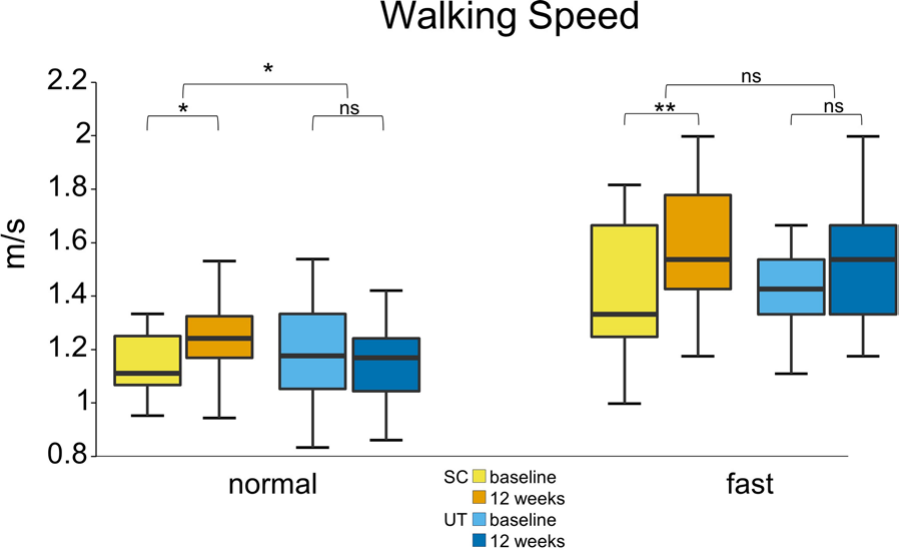
Sport climbing improves the speed of normal walking and fast walking. Box plots for each test point (before the intervention = baseline, and after the intervention = after 12 weeks) show the respective median, minimum, maximum and the first and third quartiles of the walking speed in meters per second in 22 Parkinson’s disease participants (H&Y 2–3) after a 12-week sport climbing intervention (SC) compared to a control group of 24 PD participants (H&Y 2–3) who performed unsupervised physical activity (UT). The SC showed a significant increase in speed during normal walking (baseline: 1.2 m/s, 12 weeks: 1.3 m/s, p = 0.003) and fast walking (baseline: 1.4 m/s, 12 weeks: 1.6 m/s, p = 0.001), while the UT did not (normal walking baseline: 1.2 m/s; 12 weeks: 1.2 m/s; fast walking baseline: 1.5 m/s, 12 weeks: 1.5 m/s). H&Y, Hoehn & Yahr stage; SC, sport climbing group; UT, unsupervised control group; **p ≤ 0.01. *p ≤ 0.05; n.s., not significant

Regression analysis revealed that being part of the SC significantly predicted walking speed (coeff. − 1.01; R^2^ = 0.137, p = 0.011), but not step time asymmetry. See Fig. 1A.

### Fast walking

After the intervention, the SC demonstrated an increase in walking speed by 0.1 m/s, which was significant (95% CI [0.60–0.21], p = 0.001). Additionally, step time in the SC decreased by 0.02 s (95% CI [− 0.01 to − 0.03], p = 0.002), stride time decreased by 0.05 s (95%CI [− 0.02 to − 0.07], p = 0.002), and stance time decreased by 0.03 s (95% CI [− 0.01 to − 0.05], p = 0.004).

The UT did not show significant changes in walking speed or the IMU-derived gait parameters after the intervention.

Regression analysis showed that being part of the SC predicted stance time (coeff. 0.028, R^2^ 0.092, p = 0.046), with trends toward significance for stride time (p = 0.059), and step time (p = 0.064), but it did not predict walking speed. See Fig. 1.

### Dual-task walking

After the intervention, the SC improved their cognitive single-task performance by completing it 12 s faster (95% CI [− 21.9 to − 2.4], p = 0.017), while the UT performed the motor single-task 5 s faster (95% CI [− 6.52 to − 2.98], p < 0.001).

Regression analysis did not reveal any prediction by group membership for either the cognitive or motor single-task speeds.

### Single-task vs dual-task

After the intervention, both SC and UT, there were significant decelerations in all of the dual-task gait tests compared to the single-task gait tests: comparing fast walking with motor dual-tasking, both the SC and the UT showed a reduction in walking speed by 0.3 m/s (p < 0.001) before the intervention; after the intervention, the SC demonstrated a reduction in speed by 0.4 m/s (p < 0.001) and the UT by 0.3 m/s (p < 0.001). Comparing fast walking with cognitive dual-task walking, both the SC and the UT showed a reduction of speed by 0.4 m/s (p < 0.001); after the intervention, the SC demonstrated a reduction of speed by 0.5 m/s (p < 0.001) and the UT by 0.4 m/s (p < 0.001). Comparing the 3m- TUG and the dual-task TUG, there was a reduction of speed by 0.1 m/s in the SC (p = 0.003) and the UT (p = 0.007) before the intervention. After the intervention, both the SC and the UT showed a reduction of speed by 0.1 m/s (p < 0.001). There was no significant difference between SC and UT in this respect. The differences between single-task and dual-task tasks did not significantly decrease in either SC or UT after the intervention. Regression analysis did not reveal any prediction by group membership for either the cognitive or motor task speeds.

#### ISAW

After the intervention, the SC was faster by 0.1 m/ s in the ISAW, which was significant (95% CI [0.07–0.18], p < 0.001), while the UT showed no significant improvement.

Regression analysis predicted that being part of the SC significantly predicted ISAW speed (coeff. − 0.130, R^2^ 0.119, p = 0.019).

**Fig. 2:**
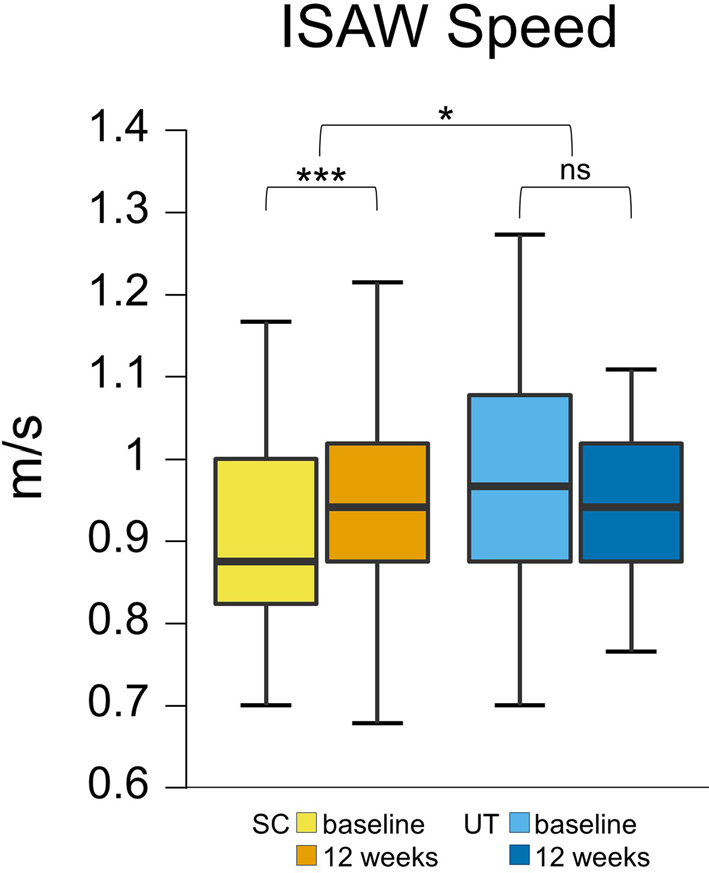
Sport climbing improves the speed of the Instrumented Stand and Walk Test. Box plots for each test point (before the intervention = baseline, and after the intervention = after 12 weeks) show the respective median, minimum, maximum and the first and third quartiles of the speed of the ISAW in meters per second in 22 Parkinson’s Disease participants (H&Y 2–3) after a 12-week sport climbing intervention (SC) compared to a control group of 24 PD participants (H&Y 2–3) who performed unsupervised physical activity (UT). The SC showed a significant increase in speed of the ISAW (baseline: 0.9 m/s, 12 weeks: 1.0 m/s, p < 0.001), while the UT did not (baseline: 1.0 m/s; 12 weeks: 1.0 m/s). H&Y, Hoehn & Yahr stage; ISAW; Instrumented Stand and Walk Test; SC, sport climbing group; UT, unsupervised control group; **p ≤ 0.01. *p ≤ 0.05; n.s., not significant

After the intervention, neither the SC nor the UT showed significant changes in IMU-derived gait parameters in the ISAW. IMU-derived gait characteristics did not significantly differ between the SC and the UT after the intervention. See Fig. 2.

#### 5TSTS

After the intervention, the SC completed the 5TSTS 2.5 s faster, which was significant (95% CI [− 4.43 to − 0.57], p = 0.014).

After the intervention, the UT did not show any significant improvement in 5TSTS speed.

**Fig. 3:**
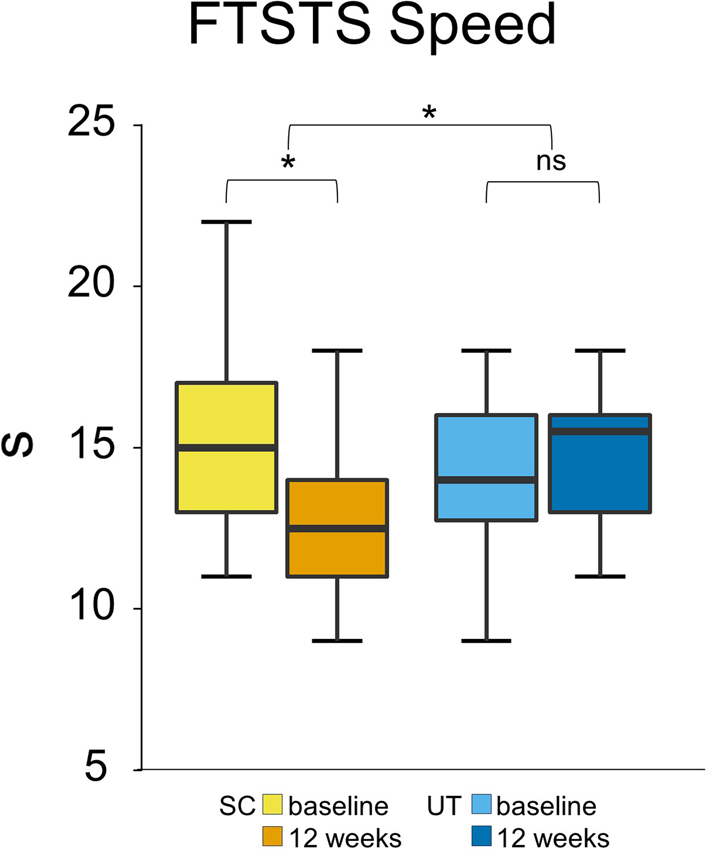
Sport climbing improves the speed of the Five Times Sit to Stand test. Box plots for each test point (before the intervention = baseline, and after the intervention = after 12 weeks) show the respective median, minimum, maximum and the first and third quartiles of the speed of the FTST in seconds in 22 Parkinson’s Disease participants (H&Y 2–3) after a 12-week sport climbing intervention (SC) compared to a control group of 24 PD participants (H&Y 2–3) who performed unsupervised physical activity (UT). The SC showed a significant increase in the speed of the FTST (baseline: 15 s, 12 weeks: 13 s, p = 0.014), while the UT did not (baseline: 15 s; 12 weeks: 15 s). H&Y, Hoehn & Yahr stage; FTST; Five Times Sit to Stand test; SC, sport climbing group; UT, unsupervised control group; **p ≤ 0.01. *p ≤ 0.05; n.s., not significant

No significant changes were observed in the IMU-derived postural transition parameters in either group. Regression analysis indicated that being part of the SC predicted the speed of the 5TSTS (coeff. 2.750, R^2^ 0.130, p = 0.014). See Fig. 3.

### TUG

#### 3 m-TUG

Neither the SC nor the UT showed a significant increase in speed in the 3m-TUG.

After the intervention, the SC showed a reduction in step time by 0.02 s (95% CI [− 0.29 to − 0.04], p = 0.011), stride time by 0.4 s (95% CI [− 0.60 to − 0.10], p = 0.008), stance time by 0.3 s (95% CI [− 0.48 to − 0.06], p = 0.013), swing time by 0.1 s (95% CI [− 0.11 to − 0.01], p = 0.017), and double limb support by 0.2 s (95% CI [− 0.19 to − 0.02], p = 0.019). No significant changes were observed in the SC for postural transition parameters.

After the intervention, there was no significant change in the IMU-derived gait- or postural transition parameters in the UT.

**Fig. 4:**
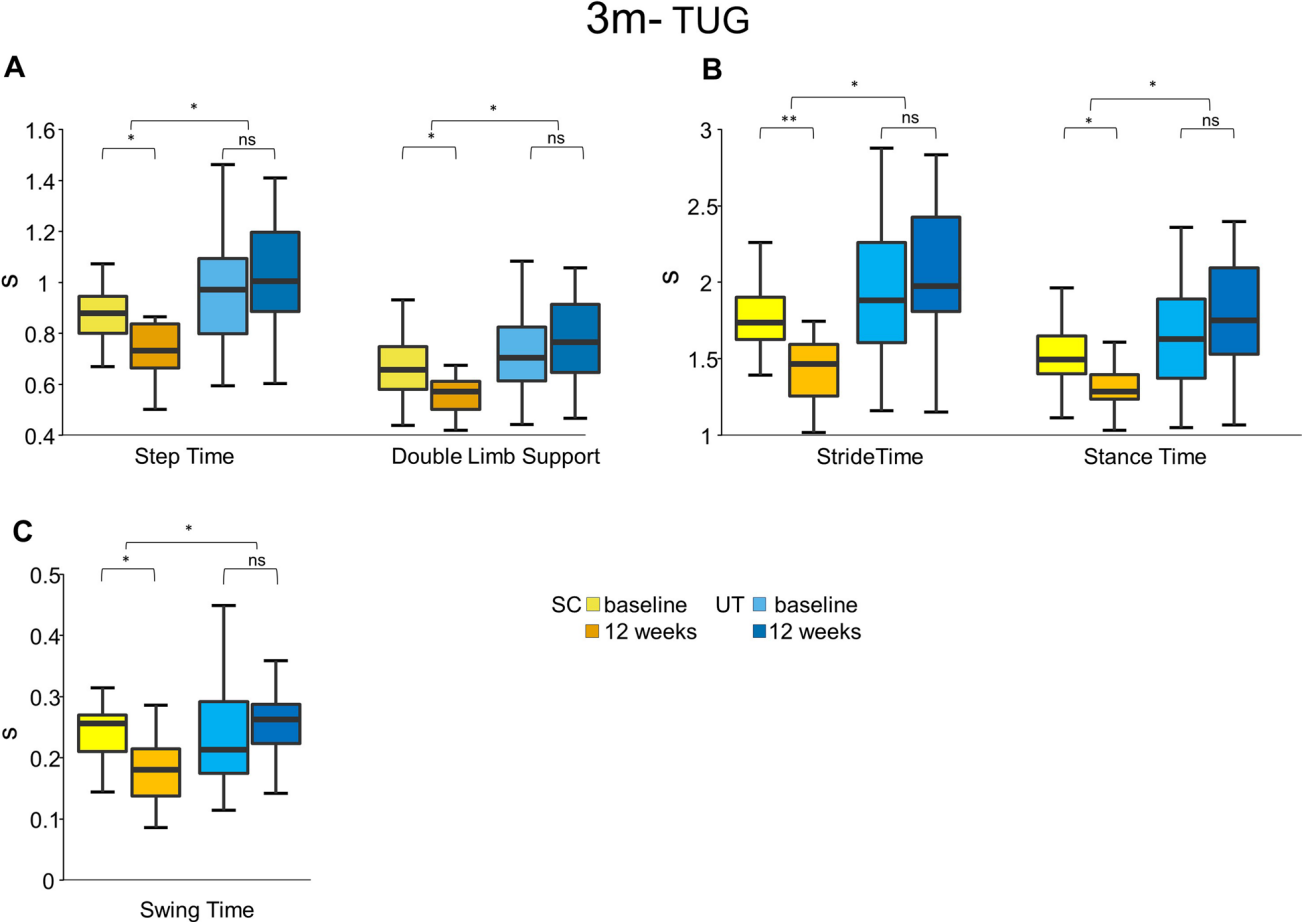
Sport climbing improves IMU-derived gait parameters during the three-meter Timed Up and Go test. Box plots for each test point (before the intervention = baseline, and after the intervention = after 12 weeks) show the respective median, minimum, maximum and the first and third quartiles of the IMU-derived gait parameters during the 3m-TUG in seconds in 22 Parkinson’s Disease participants (H&Y 2–3) after a 12-week sport climbing intervention (SC) compared to a control group of 24 PD participants (H&Y 2–3) who performed unsupervised physical activity (UT). The SC showed a significant increase in Step Time (baseline: 1 s, 12 weeks: 0.8 s, p = 0.011), Double Limb Support (baseline: 0.7 s, 12 weeks: 0.6 s, p = 0.019) (**A**), Stride Time (baseline: 1.9 s, 12 weeks: 1.5 s, p = 0.008), Stance Time (baseline: 1.6 s, 12 weeks: 1.3 s, p = 0.013) (**B**), and Swing Time (baseline: 0.3 s, 12 weeks: 0.2 s, p = 0.017) (**C**). The UT did not show any increase in Step Time (baseline: 1 s, 12 weeks: 1 s), Double Limb Support (baseline: 0.7 s, 12 weeks: 0.8 s), Stride Time (baseline: 2 s, 12 weeks: 2 s), Stance Time (baseline: 1.7 s, 12 weeks: 1.8 s), Swing Time (baseline: 0.3 s, 12 weeks: 0.3 s). 3 m-TUG, three-meter Timed Up and Go test; SC, sport climbing group; UT, unsupervised control group; **p ≤ 0.01. *p ≤ 0.05; n.s., not significant

Regression analysis revealed that being part of the SC predicted step time (coeff. 0.231, R^2^ 0.360, p = 0.018), stride time (coeff. 0.463, R^2^ 0.118, p = 0.024), stance time (coeff. 0.398, R^2^ 0.130, p = 0.018), swing time (coeff.0.081, R^2^ 0.140, p = 0.013), and double limb support (coeff.0.150, R^2^ 0.097, p = 0.042) of the 3 m-TUG. See Fig. 4.

#### 7 m-TUG

After the intervention, the SC exhibited an increase in speed by 0.1 m/s (95% CI [− 0.15 to − 0.02], p = 0.011), which was significant; there was also a trend toward significance in the duration of turns (p = 0.052).

The UT did not significantly improve the speed of the 7 m-TUG; they showed a reduction of the number of steps by 0.7 steps/m (95% CI [− 1.26 to − 0.08], p = 0.027) and an increase, i.e. worsening of step time by 0.1 s (95% CI [0.02 to 0.21], p = 0.015), stride time by 0.2 s (95% CI [0.04 to 0.42], p = 0.022), stance time by 0.2 s (95% CI [0.03 to 0.34], p = 0.019), and double limb support by 0.1 s (95% CI [0.01 to 0.14], p = 0.026); there was no significant change in the IMU-derived postural transition parameters in the UT.

Regression analysis indicated that being part of the SC predicted the duration of turns in the 7 m-TUG (coeff. 0.646, R^2^ 0.080, p = 0.02), but not speed or any of the IMU-derived gait- or postural transition characteristics.

#### Dual-task TUG

Neither the SC nor the UT showed a significant increase in speed in the dual-task TUG.

## Discussion

This secondary analysis of a randomized controlled trial aimed to compare the effects of sport climbing versus unsupervised physical exercise on gait and functional mobility parameters in PD participants. The main findings were that sport climbing improved gait speed during normal walking, stance time during fast walking, speeds of the ISAW and the 5TSTS, and turning speed of the 7 m-TUG, as well as step time, stride time, stance time, swing time, and double limb support of the 3 m-TUG. Neither SC nor UA improved their performance during the dual-task assessments. These results suggest that movement training in the vertical plane, as is the case in sport climbing, has a discernible impact on mobility in the horizontal plane.

### Normal gait

The increase in normal gait speed in SC could be explained, at least partly, by the fact that sport climbing reduces bradykinesia [47, 70]. The increase in speed did not occur at the cost of cadence or step length, which remained unchanged during both normal and fast walking. In PD, a decrease in step length is most likely the result of impaired executive function of the supplementary cortex caused by the degeneration of the basal ganglia [71, 72]. Gait hypometria in PD impedes an increase in step length to achieve faster speeds [73, 74]; instead, the cadence increases during fast walking as a compensatory mechanism [75]. Sport climbing appeared to at least partly counteract this pathological compensatory gait pattern insofar as it allowed the participants of the climbing group to walk faster without sacrificing step length. The increase in speed coupled with a stable—albeit not outright improved—step length is comparable to previous research on gait-focused exercises such as treadmill training [76–78], and gait-targeted physical therapy [25, 79, 80].

We detected a beneficial effect of sport climbing on step time symmetry during normal walking, and a reduction in step time, stride time, and stance time during fast walking (although only the between-group differences in stance time were large enough to reach statistical significance). This suggests that sport climbing could lead to a more balanced, symmetrical, more efficient and faster gait pattern. These findings are basically in line with previous studies on non-gait-focused physiotherapy, that found an increase in walking speed, but no significant effects on other IMU-derived gait parameters, such as step length, cadence, double limb support variability, step time, stride time, stance time, swing time, double limb support, asymmetry, and step time variability [27, 32, 81–85].

In summary, although sport climbing had a pronounced effect on gait speed, the beneficial effect on specific gait variables as observed after gait-specific exercises [25, 77, 80, 86] may exist but larger cohorts may be needed to investigate this in more detail. While climbing is a highly challenging sport regarding movement planning, hand–eye coordination, and spatial awareness [87, 88], it does obviously not train the repetitive smooth movements that make up a physiological gait pattern in the same way as gait-focused training. Thus, our study suggests that sport climbing is well suited for PD participants with decreased walking speed, while PD participants who exhibit marked Parkinsonian gait disorder may require specifically targeted physiotherapy.

Notably, the gain of speed did not come at the expense of balance, symmetry, or rhythm, as the latter parameters did not change due to the intervention. None of the previously reported fall risk-associated parameters –stride time [89], stance time [90], double limb support [91], asymmetry [92], and step time variability [93] deteriorated significantly with SC. From this, we can conclude that SC increases the walking speed of PD participants without sacrificing safety.

### ISAW, 5TSTS, and TUG

The SC, but not the UT, walked significantly faster during the 5TSTS and the 7 m-TUG and turned faster in the latter. The SC also showed significant improvement in the step time, stride time, stance time, swing time and double limb support during the 3 m-TUG.

The TUG, the ISAW, and the 5TSTS measure mobility aspects including, but also going beyond gait [61, 85, 94, 95]. They are a compound measure of bradykinesia, balance, gait performance, and leg strength [64, 96], and are linked to overall physical fitness in PD [64, 85, 95, 97]. Deficits in these functional mobility aspects may substantially translate to decreased independence in daily life as well as to a higher fall risk [96]. Conventional physiotherapy [98], resistance training [70], balance training [85], dancing [27, 35], aquatic exercise [36] and even an intensive walking regime [99] have been shown to improve performance in these tests. Our study is the first to show an effect of sport climbing on these scores, indicating enhanced gait coordination, gait efficiency, and lower limb strength. We hypothesize that the main driver of these results may be the already observed reduction in bradykinesia [47]. SC is also known to increase the strength in the lower extremities [39, 100], which could additionally improve these mobility aspects. However, we did not formally test lower extremity strength in our study. In summary, we demonstrated that sport climbing as a kind of vertical mobility translates well to better mobility on the ground, indicating that functional mobility aspects, beyond gait, are also positively influenced by this training.

The SC had a climbing session only once a week but still showed superior benefit compared to those performing the longer unsupervised exercise sessions. It is therefore reasonable to argue that climbing is far more efficient than unsupervised training in promoting gait speed and further mobility aspects.

### Dual-task walking

During dual-task walking, the IMU-derived gait parameters did not improve in the SC. It appears that even though climbing trains complex movement patterns [87, 88], it does not relevantly influence dual-task gait in the conditions tested with our study protocol. Similar to the single-task gait discussed above, climbing appeared to have less of an impact on gait patterns than gait-specific exercise [101, 102] and dual-task-gait-targeted exercise [34, 103, 104], which can improve dual-tasking abilities. As a result, sport climbing appears to be less ideal for PD participants with dual-tasking issues, although further research is needed.

It is important to note that there was no deterioration of accelerometric gait parameters under dual-task conditions compared to single-task conditions in either group even at baseline. This contradicts prior findings that dual-task conditions worsen IMU-derived gait characteristics [80]. This lack of discernible difference in the IMU-derived gait parameters between single- and dual-task conditions even at baseline could also explain why there was no measurable effect after the intervention.

### Clinical implications

Sport climbing is effective in enhancing single-task gait speed and functional mobility. Thus, sport climbing can be a viable option for individuals with PD who experience deficits in this area searching for an enjoyable activity that can help maintain their mobility and independence.

### Limitations of the study

We recognize some potential limitations of the study. First, our follow-ups were conducted over the phone, so follow-up testing of gait and functional mobility was not possible. Second, we recruited mild to moderately affected PD participants, omitting an analysis of the effects of climbing on PD participants at either extreme of the Hoehn & Yahr scale. Minimally affected PD participants could not benefit from climbing in terms of gait, while climbing might prove too difficult for severely affected PD participants to be a valid therapeutic option. However, climbing could be even more effective in the early stages of PD because participants are fitter. It is also worth noting that the 7-m TUG, in contrast to the 3-m TUG, has not been validated, which limits the conclusions that can be drawn from it. Another important limitation is the small sample size and the fact that for this secondary analysis of a randomized controlled trial, there was no formal sample size calculation for this comparison.

## Conclusion

To the best of our knowledge, this is the first randomized controlled trial to compare sport climbing with an active control group investigating gait and functional mobility. Sport climbing significantly improved gait speed and functional mobility in PD participants, suggesting that vertical plane training has the potential to improve horizontal plane mobility. As qualitative gait parameters, such as variability and symmetry, did not worsen in the course of the intervention, we argue that climbing is a promising sport to improve gait and functional mobility in PD participants. Further studies are needed to assess the long-term effects and feasibility of sport climbing in PD participants.

### Supplementary Information

**Additional file 1:** Inertial measurement unit-derived gait parameters.** Table S1.** shows the mean values, standard deviation (SD) and the mean changes with 96% confidence intervals (95% CI) for gait variables collected during normal and fast walking, dual-task walking, and functional mobility tests at baseline and after the intervention.

## Data Availability

The datasets used and/or analyzed during the current study are available from the corresponding author upon reasonable request.

## Abbreviations

5TSTS: Five times sit to stand test
H&Y: Hoehn & Yahr stage
IMU: Inertial measurement unit
ISAW: Instrumented stand and walk test
MDS-UPDRS III: Movement Disorder Society-Sponsored Revision of the Unified Parkinson’s Disease Rating Scale part III
MMSE: Mini Mental State Examination
PD: Parkinson’s disease
SC: Sport climbing group
TUG: Timed up and go test
UT: Unsupervised training group
WHO: World Health Organization
